# Chondroid Syringoma of the Medial Canthus

**DOI:** 10.1155/2014/158527

**Published:** 2014-03-11

**Authors:** Konstantinos Paraskevopoulos, Angeliki Cheva, Giorgos Koloutsos, Ioannis Matzarakis, Kostas Vahtsevanos

**Affiliations:** ^1^Department of Oral and Maxillofacial Surgery, General Hospital of G. Papanikolaou, 57010 Thessaloniki, Greece; ^2^Department of Pathology, General Hospital of G. Papanikolaou, 57010 Thessaloniki, Greece

## Abstract

Chondroid syringoma, or pleomorphic adenoma of the skin, is a rare, benign skin adnexal tumor. It is usually exhibited as a slowly growing intradermal or subcutaneous nodule, typically located in the head and neck region. Because of the unremarkable clinical symptomatology of this rare tumor, the diagnosis is exclusively made retrospectively based on histological confirmation of the surgically excised tumor. We present a rare case of a chondroid syringoma located in the left medial canthus of a 58-year-old man. The patient had no symptoms and decided to excise it for cosmetic reasons only. Local excision with a macroscopic wide cuff of normal tissue was done, without destroying the aesthetic and functional structures, since the defect was restored by a finger flap. One year postoperatively, the patient has remained disease-free. Chondroid syringoma usually appears in the face but the location in the medial canthus is not mentioned in the literature for the last twenty years.

## 1. Introduction

The chondroid syringoma, or mixed tumor of the skin, is a benign neoplasm of sweat gland origin and a rare clinical entity. Histologically, it could be described as a neoplasm consisting of epithelial and myoepithelial cell formations within a myxomatous, pseudochondromatous, or hyaline stroma that contains mucous secretions [1]. It is usually located in the skin of the face and the scalp in patients between the age of 20 and 60 years with a distinct male predominance [2]. The most common sites are the nose, cheek, upper lip, scalp, forehead, and chin [3]. Chondroid syringoma appears clinically as a slowly growing, painless or subcutaneous nodule. Lesions are firm and adherent to overlying skin but distinct from underlying structures.

We report a very rare case of chondroid syringoma located in the left medial canthus. This location has not been mentioned in the literature for the last twenty years.

## 2. Case Report

A 53-year-old male patient consulted our oral and maxillofacial surgery outpatient clinic for an asymptomatic exophytic lesion in the left medial canthus. The patient requested surgery for cosmetic reasons.

According to the patient the lesion had first appeared six months before the clinical examination and it was growing very slowly. He did not complain of any symptoms except for the cosmetic problems.

**Figure 1:**
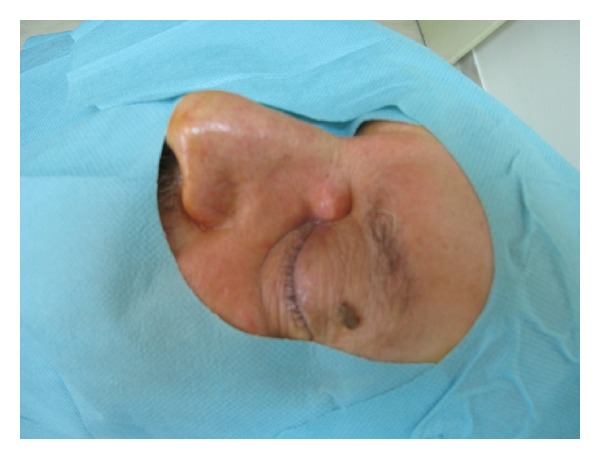
The lesion in the left medial canthus.

On examination the lesion diameter was 1 cm and located in his left medial canthus, without limiting his sight (Figure 1).

**Figure 2:**
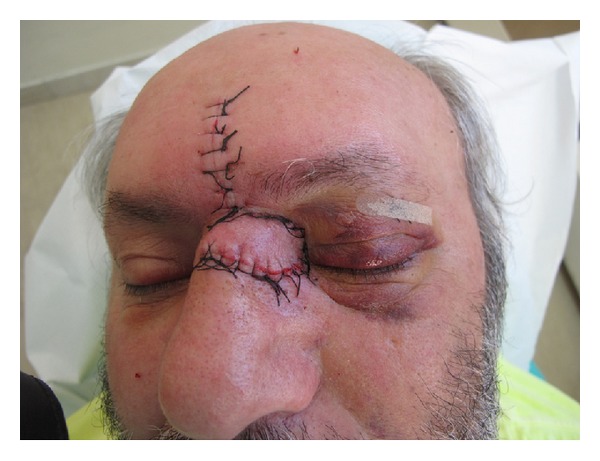
Reconstruction with glabellar flap.

Under local infiltration anesthesia the lesion was excised and sent for histopathological examination. Reconstruction was carried out with a classic glabellar flap (Figure 2).

Healing was uneventful and the patient was satisfied with the cosmetic result. One year later there is no recurrence.

**Figure 3:**
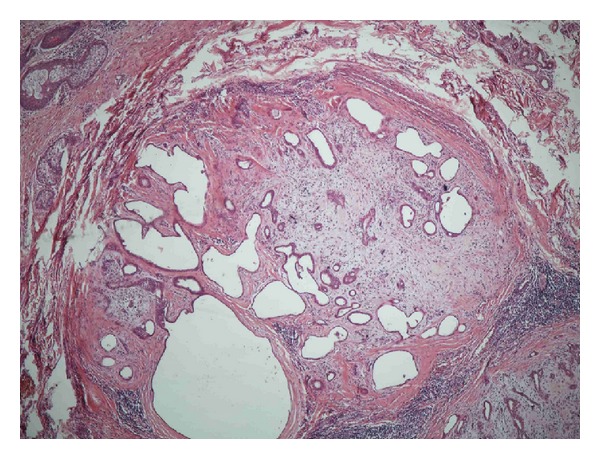
Multinodular lesion.

**Figure 4:**
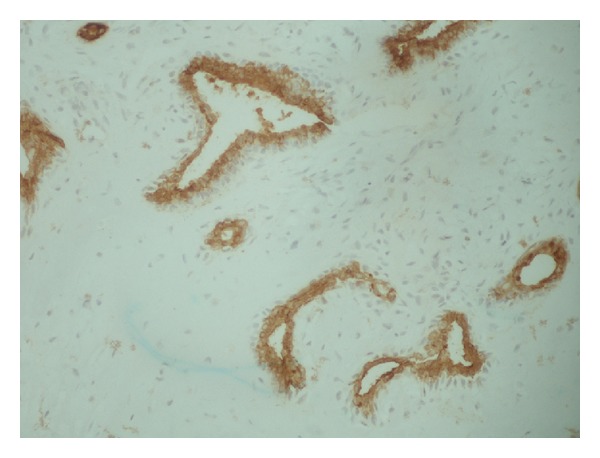
ck7 epithelial cells ×200.

**Figure 5:**
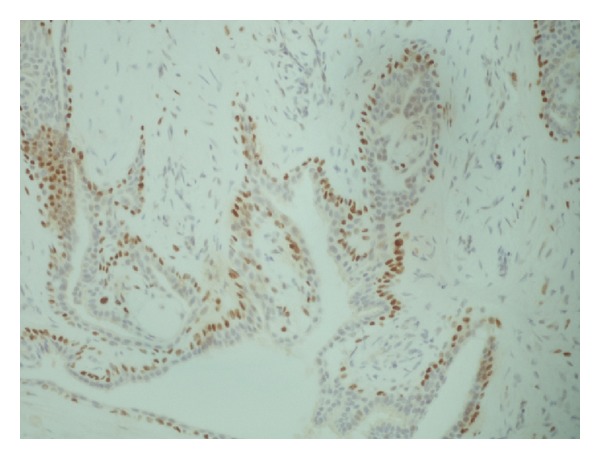
p63 myoepithelial cells ×200.

**Figure 6:**
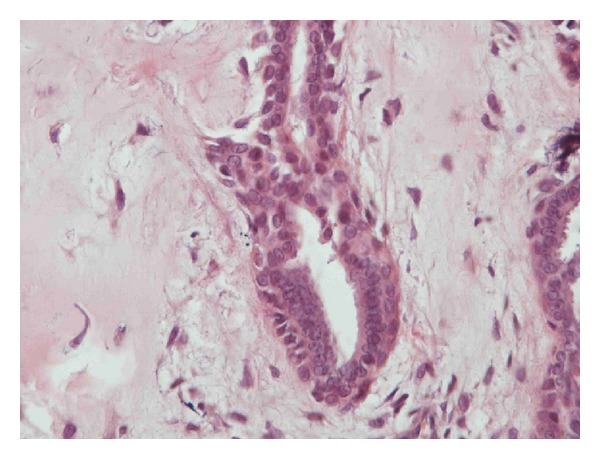
myoepithelial cells.

**Figure 7:**
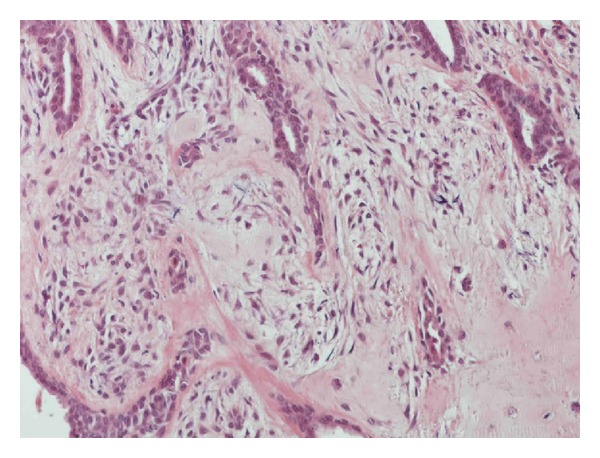
Myxoid mesenchymal matrix.

**Figure 8:**
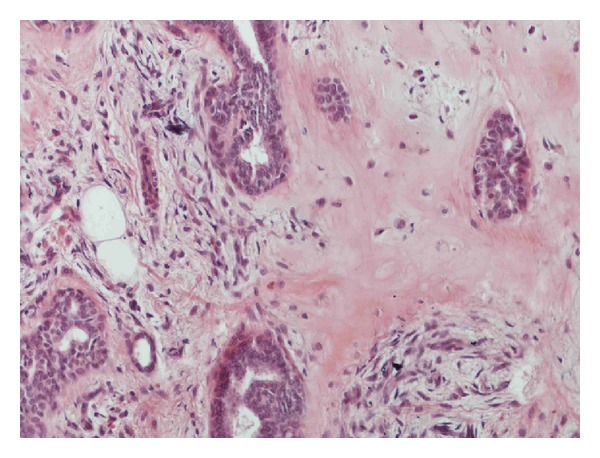
Hyaline mesenchymal matrix.

The histological examination revealed a circumscribed, multinodular lesion (Figure 3), located in the dermis of the eyelid. The tumor consisted of nests and tubules of epithelial (Figure 4) and myoepithelial cells (Figures 5 and 6), in myxoid (Figure 7) and hyaline (Figure 8) mesenchymal matrix.

No atypia or unusual mitotic activity was observed. The diagnosis of pleomorphic adenoma (mixed tumor) was performed.

## 3. Discussion

Sweat gland tumors in the head and neck are uncommon. Chondroid syringoma, which was first described by Hirsch and Helwig in 1961 [3], is a rare benign mixed tumor of the sweat glands localized in the dermis or subdermis. It occurs most frequently in the head and neck and the commonest sites are scalp, cheek, nose, upper lip, chin, and forehead. Less commonly this tumor can involve hand, foot, axillary region, abdomen, penis, vulva, and scrotum [19, 21–26]. Chondroid syringoma usually affects middle aged male patients over 35 years of age [2, 27].

We report a rare case of chondroid syringoma located in medial canthus, which has not been mentioned in the literature (Table 1).

The lesion is a slow-growing, painless, intradermal, or subcutaneous nodule and may be attached to the overlying skin with no fixation to deeper structures [28]. The diagnosis is usually made retrospectively based on histopathological findings. The clinical diagnosis is confirmed by the histological examination of the lesion. A clinical differential diagnosis may include implantation dermoid, sebaceous cyst, compound naevus, clear cell hidradenoma, cystic basal cell carcinoma, neurofibroma, and dermatofibroma. The deep variant of this tumour could be confused with a pleomorphic adenoma of major or minor salivary gland origin [29].

Treatment of choice is local surgical excision with a cuff of normal tissue, in order to prevent recurrence. If the tumor has been completely excised and is benign, long-term followup is not indicated. Followup is indicated only if the excision is incomplete or if there is indication of malignant change, which is rare but has been reported in the literature [17].

## 4. Conclusion 

Chondroid syringoma is an uncommon mixed tumor of the skin, which usually appears on the face. Maxillofacial surgeons must be aware of these tumors, as they can easily be misdiagnosed, because of the rare occurrence of them. However, it should be included in the differential diagnosis of any slowly growing nodule in the skin of the face. The treatment of choice is local excision. Recurrence is attributed to incomplete excision or malignant transformation, which, although being rare, has been reported.

## Figures and Tables

**Table 1:** Location of the reported cases the last 20 years

Location	Number	References
Cheek	1	[4]
Forehead	1	[5]
Scalp	4	[6–8]
Upper lip	2	[6, 9]
Nose	2	[10, 11]
Eyelid	2	[12]
Nasofacial groove	1	[13]
Philtrum	1	[14]
Ear	4	[15–18]
Orbit	2	[19, 20]
Medial canthus	1	Our case

## References

[1] Wetli CV, Pardo V, Millard M, Gerston K (1972). Tumors of ceruminous glands. *Cancer*.

[2] Stout AP, Gorman JG (1959). Mixed tumors of skin of thesalivary gland type. *Cancer*.

[3] Hirsch P, Helwig EB (1961). Chondroid syringoma. Mixed tumor of skin, salivary gland type. *Archives of Dermatology*.

[4] Awasthi R, Harmse D, Courtney D, Lyons CBA (2004). Benign mixed tumour of the skin with extensive ossification and marrow formation: a case report. *Journal of Clinical Pathology*.

[5] Agrawal A, Kumar A, Sinha AK, Kumar B, Sabira KC (2008). Chondroid syringoma. *Singapore Medical Journal*.

[6] Dubb M, Michelow P (2010). Cytologic features of chondroid syringoma in fine needle aspiration biopsies: a report of 3 cases. *Acta Cytologica*.

[7] Paik YS, Liess BD (2011). Chondroid syringoma of the scalp: case report and discussion of clinical features, histopathology, and treatment. *Ear, Nose and Throat Journal*.

[8] Schiano di Visconte M, Picciano P (2002). Chondroid syringoma. A case report. *Chirurgia Italiana*.

[9] Arikan OK, Erdoğan S, Muluk NB, Koç C (2004). Chondroid syringoma of the upper lip: a case report. *Kulak Burun Bogaz Ihtisas Dergisi*.

[10] Kumar B (2010). Chondroid syringoma diagnosed by fine needle aspiration cytology. *Diagnostic Cytopathology*.

[11] Bhargava D, Bhusnurmath S, Daar AS (1997). Chondroid syringoma of the nose: report of a case. *Journal of Laryngology and Otology*.

[12] Mencía-Gutiérrez E, Bonales-Daimiel JA, Gutiérrez-Díaz E, Santos-Briz A, Madero-García S (2001). Chondroid syringomas of the eyelid: two cases. *European Journal of Ophthalmology*.

[13] Chen AH, Moreano EH, Houston B, Funk GF (1996). Chondroid syringoma of the head and neck: clinical management and literature review. *Ear, Nose and Throat Journal*.

[14] Tokyol C, Aktepe F, Yavas BD, Yildiz H, Aycicek A (2010). Chondroid syringoma: a case report. *Acta Cytologica*.

[15] Kaushik V, Bhalla RK, Nicholson C, de Carpentier JP (2005). The chondroid syringoma: report of a case arising from the external auditory canal. *European Archives of Oto-Rhino-Laryngology*.

[16] Karnwal A, Pakalapati S, Tzifa K, Raut V (2006). Chondroid syringoma of the external ear canal presenting as a cyst. *Kulak Burun Bo*¿*az Ihtisas Dergisi*.

[17] Vasileiadis I, Kapetanakis S, Petousis A, Karakostas E, Simantirakis C (2011). Rapidly growing chondroid syringoma of the external auditory canal: report of a rare case. *Case Reports in Medicine*.

[18] Markou K, Karasmanis I, Vlachtsis K, Petridis D, Nikolaou A, Vital V (2008). Primary pleomorphic adenoma of the external ear canal. Report of a case and literature review. *American Journal of Otolaryngology*.

[19] Kitazawa T, Hataya Y, Matsuo K (1999). Chondroid syringoma of the orbit. *Annals of Plastic Surgery*.

[20] Belfquih H, Elmostarchid B, Oukabli M, Akhaddar A, Boucetta M (2012). Benign chondroid syringoma of the orbit: a rare cause of exophtalmos. *Head & Face Medicine*.

[21] Yavuzer R, Başterzi Y, Sari A, Bir F, Sezer C (2003). Chondroid syringoma: a diagnosis more frequent than expected. *Dermatologic Surgery*.

[22] Bekerecioglu M, Tercan M, Karakok M, Atik B (2002). Benign chondroid syringoma: a confusing clinical diagnosis. *European Journal of Plastic Surgery*.

[23] Hardisson D, Linares MD, Nistal M (1998). Giant chondroid syringoma of the axilla. *Journal of Cutaneous Medicine and Surgery*.

[24] Sliwa-Hähnle K, Obers V, Lakhoo M, Saadia R (1996). Chondroid syringoma of the abdominal wall: a case report and review of the literature. *South African Journal of Surgery*.

[25] Poku JW, Sant GR, Ucci AA (1996). Chondroid syringoma of the scrotum. *Journal of International Medical Research*.

[26] Nemoto K, Kato N, Arino H (2002). Chondroid syringoma of the hand. *Scandinavian Journal of Plastic and Reconstructive Surgery and Hand Surgery*.

[27] Ashley DJB (1978). *Evans Histological Appearrence of Tumours*.

[28] Lever WF (1983). *Histopathology of the Skin*.

[29] Gottschalk-Sabag S, Glick T (1994). Chondroid syringoma diagnosed by fine-needle aspiration: a case report. *Diagnostic Cytopathology*.

